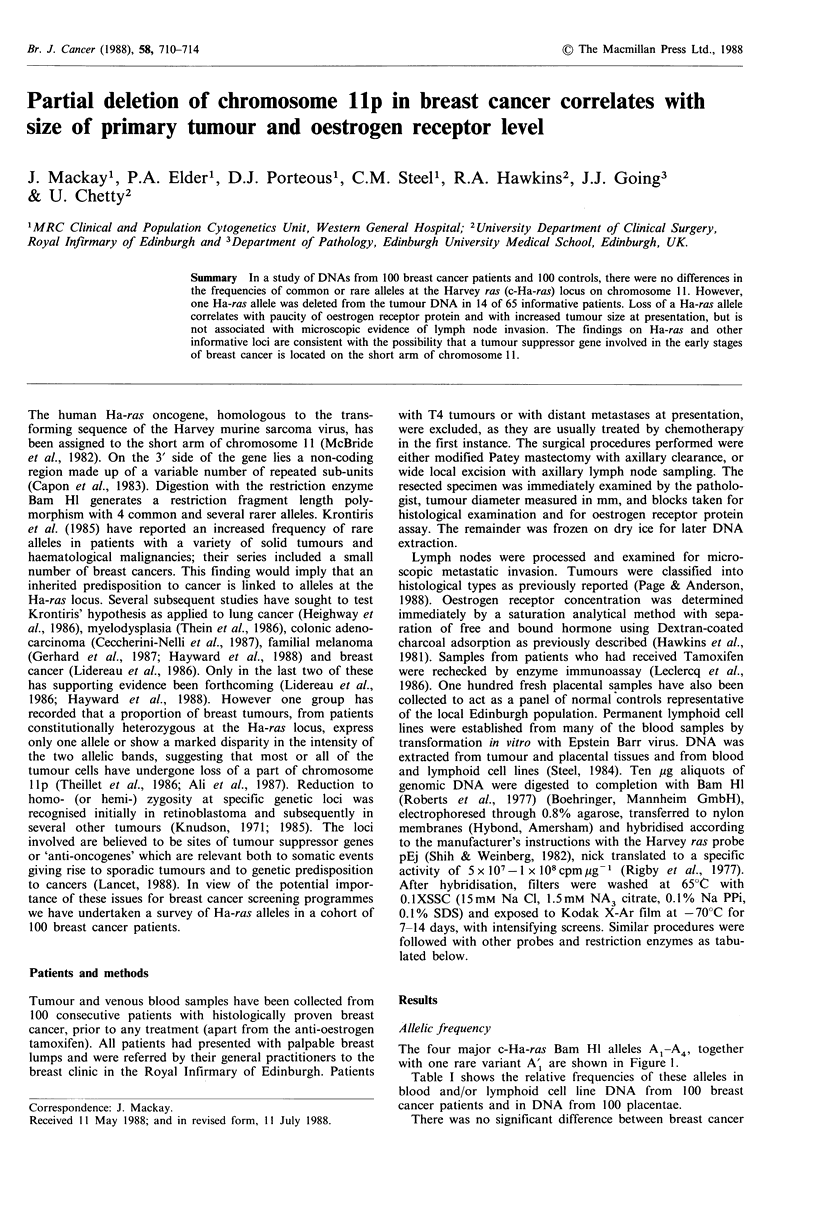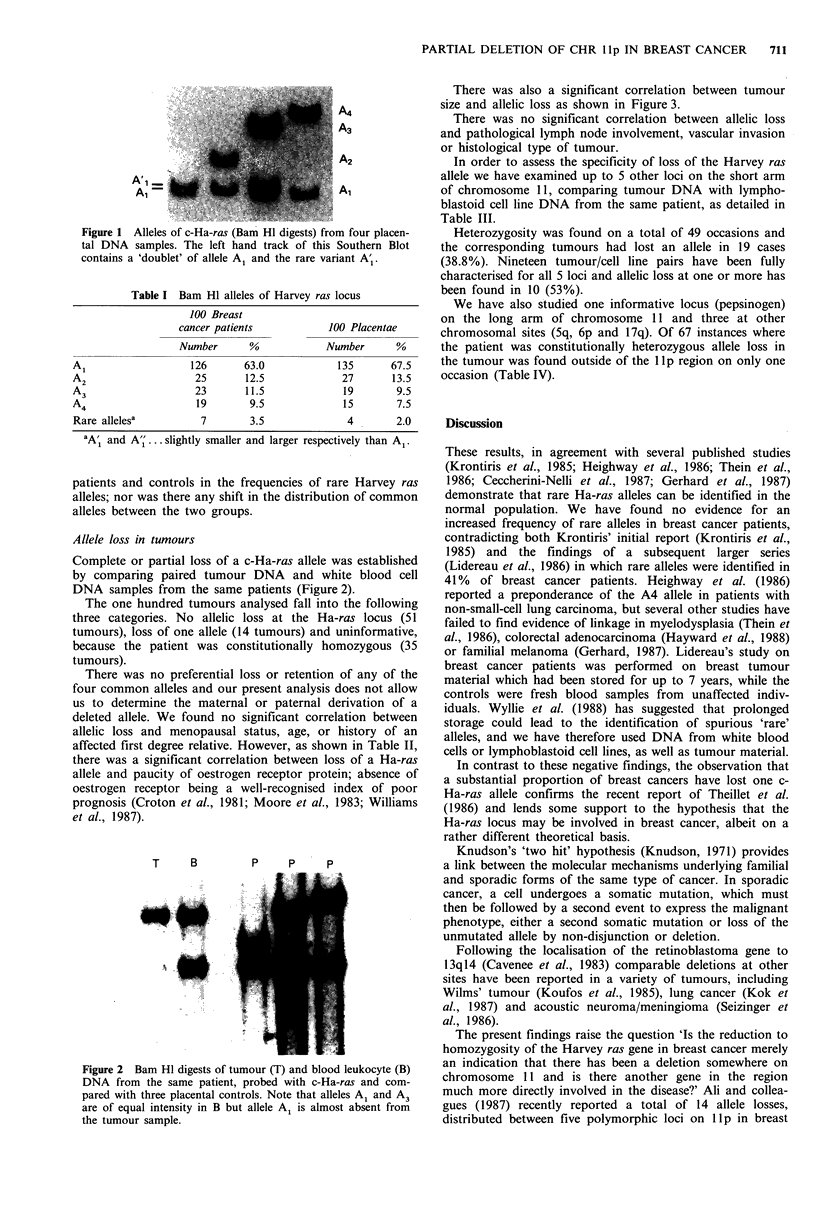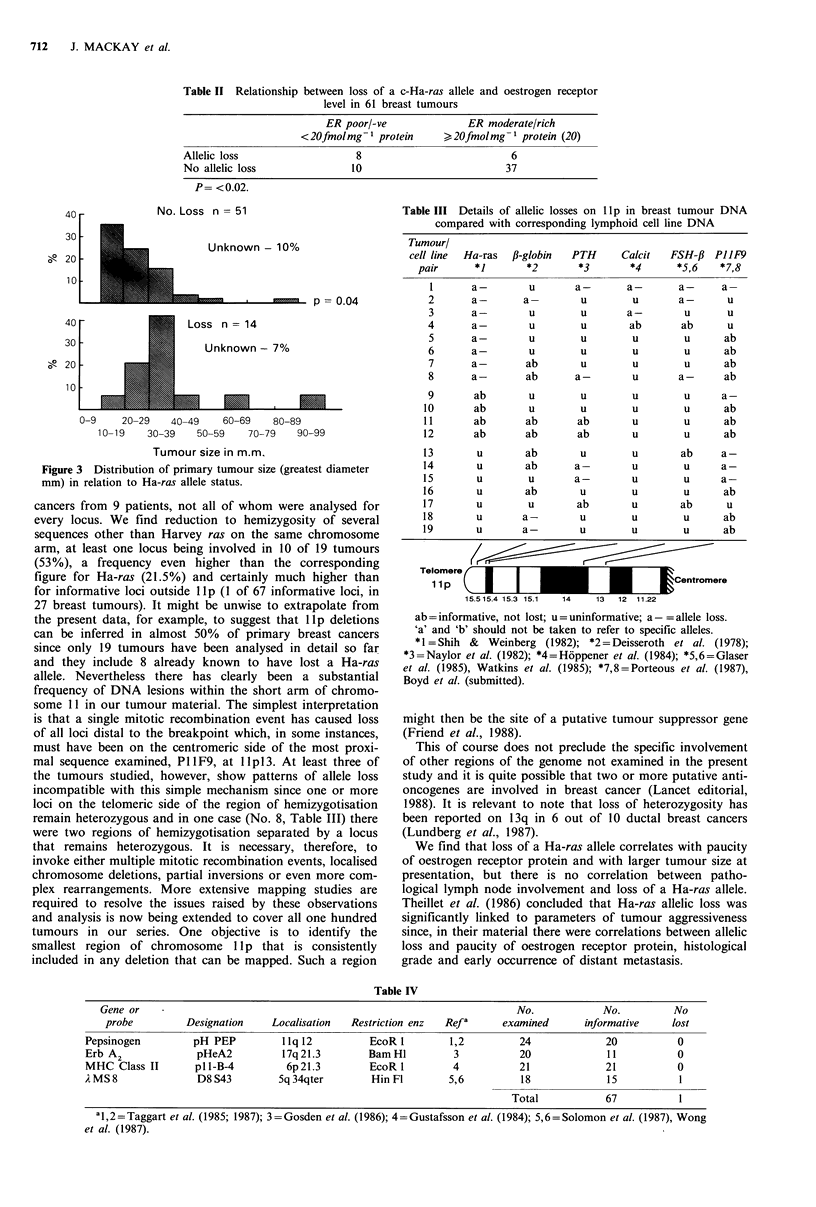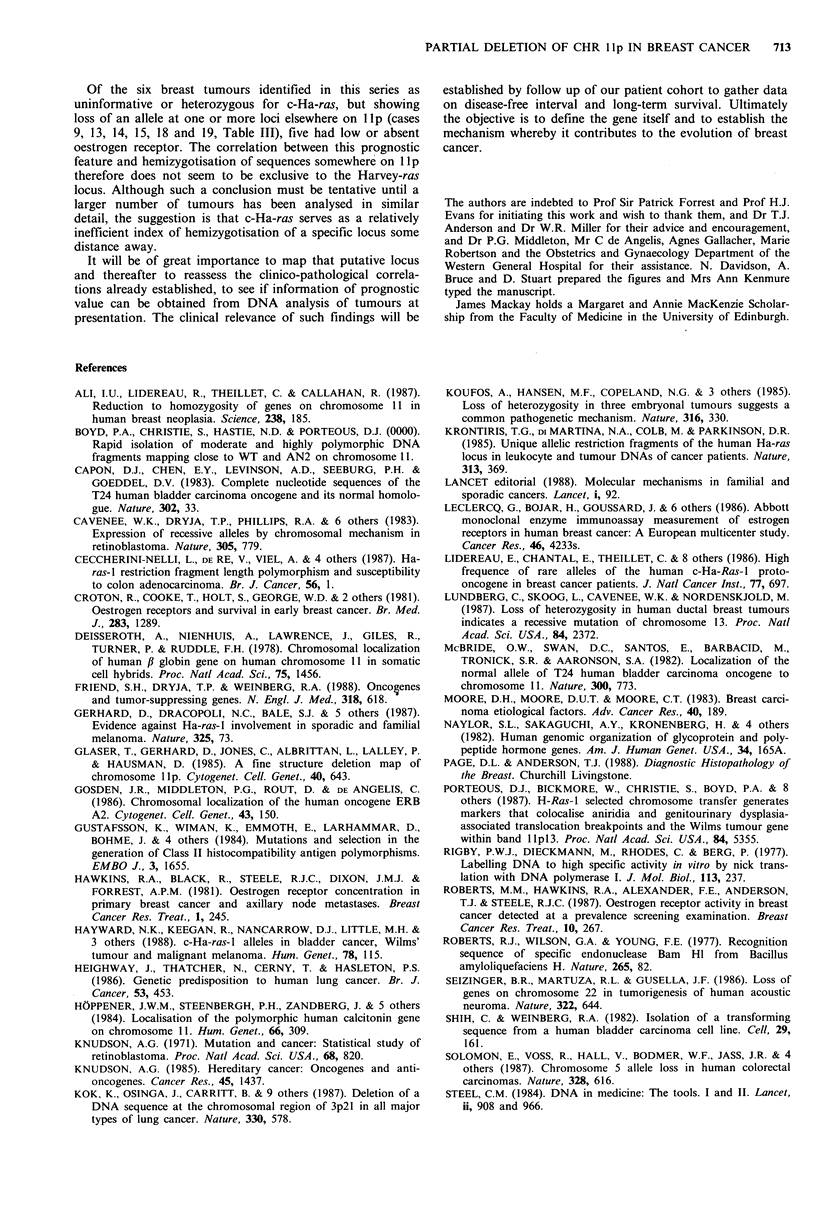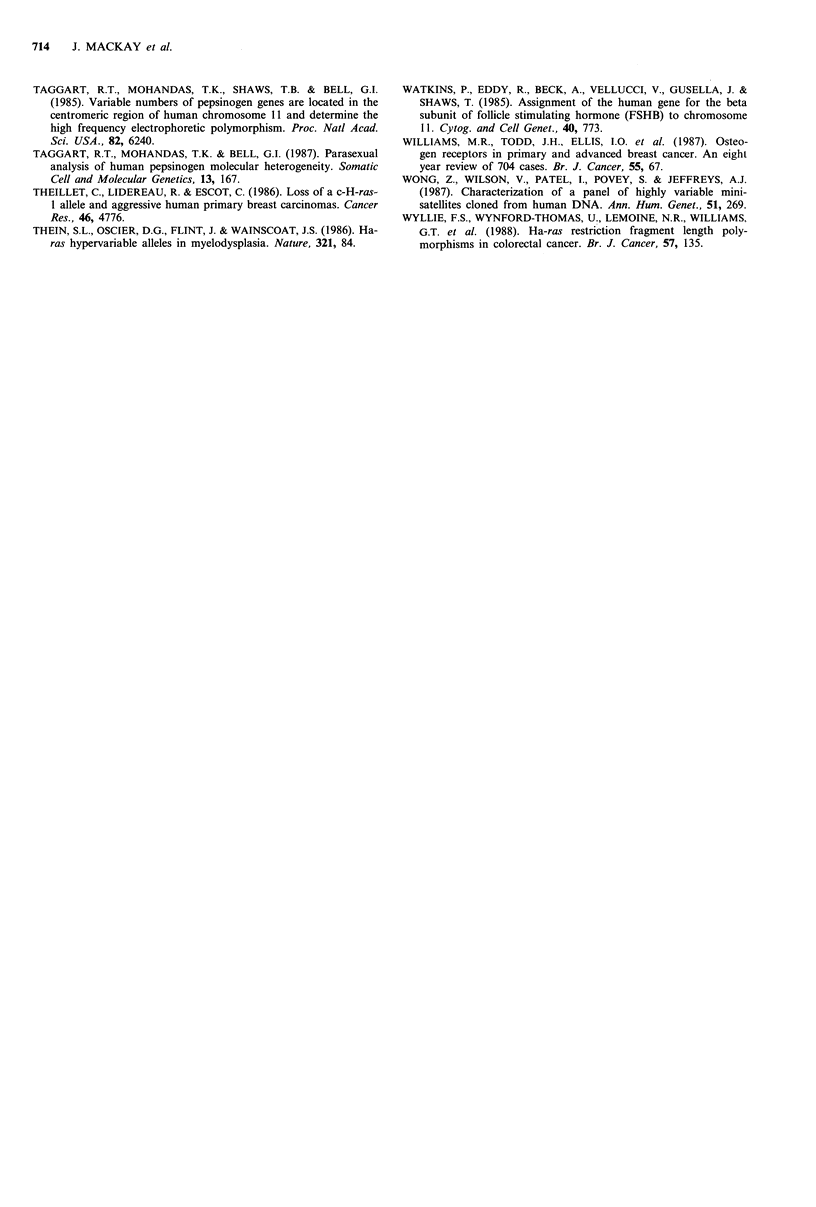# Partial deletion of chromosome 11p in breast cancer correlates with size of primary tumour and oestrogen receptor level.

**DOI:** 10.1038/bjc.1988.295

**Published:** 1988-12

**Authors:** J. Mackay, P. A. Elder, D. J. Porteous, C. M. Steel, R. A. Hawkins, J. J. Going, U. Chetty

**Affiliations:** MRC Clinical and Population Cytogenetics Unit, Western General Hospital, Edinburgh, UK.

## Abstract

**Images:**


*Br. J. Cancer* (1988), **58**, 710–714 © The Macmillan Press Ltd., 1988

### Partial deletion of chromosome 11p in breast cancer correlates with size of primary tumour and oestrogen receptor level

J. Mackay[1], P.A. Elder[1], D.J. Porteous[1], C.M. Steel[1], R.A. Hawkins[2], J.J. Going[3] & U. Chetty[2]

[1]*MRC Clinical and Population Cytogenetics Unit, Western General Hospital;* [2]*University Department of Clinical Surgery, Royal Infirmary of Edinburgh and* [3]*Department of Pathology, Edinburgh University Medical School, Edinburgh, UK.*

**Summary** In a study of DNAs from 100 breast cancer patients and 100 controls, there were no differences in the frequencies of common or rare alleles at the Harvey *ras* (c-Ha-*ras*) locus on chromosome 11. However, one Ha-*ras* allele was deleted from the tumour DNA in 14 of 65 informative patients. Loss of a Ha-*ras* allele correlates with paucity of oestrogen receptor protein and with increased tumour size at presentation, but is not associated with microscopic evidence of lymph node invasion. The findings on Ha-*ras* and other informative loci are consistent with the possibility that a tumour suppressor gene involved in the early stages of breast cancer is located on the short arm of chromosome 11.

The human Ha-*ras* oncogene, homologous to the transforming sequence of the Harvey murine sarcoma virus, has been assigned to the short arm of chromosome 11 (McBride *et al.*, 1982). On the 3′ side of the gene lies a non-coding region made up of a variable number of repeated sub-units (Capon *et al.*, 1983). Digestion with the restriction enzyme Bam Hl generates a restriction fragment length polymorphism with 4 common and several rarer alleles. Krontiris *et al.* (1985) have reported an increased frequency of rare alleles in patients with a variety of solid tumours and haematological malignancies; their series included a small number of breast cancers. This finding would imply that an inherited predisposition to cancer is linked to alleles at the Ha-*ras* locus. Several subsequent studies have sought to test Krontiris' hypothesis as applied to lung cancer (Heighway *et al.*, 1986), myelodysplasia (Thein *et al.*, 1986), colonic adenocarcinoma (Ceccherini-Nelli *et al.*, 1987), familial melanoma (Gerhard *et al.*, 1987; Hayward *et al.*, 1988) and breast cancer (Lidereau *et al.*, 1986). Only in the last two of these has supporting evidence been forthcoming (Lidereau *et al.*, 1986; Hayward *et al.*, 1988). However one group has recorded that a proportion of breast tumours, from patients constitutionally heterozygous at the Ha-*ras* locus, express only one allele or show a marked disparity in the intensity of the two allelic bands, suggesting that most or all of the tumour cells have undergone loss of a part of chromosome 11p (Theillet *et al.*, 1986; Ali *et al.*, 1987). Reduction to homo- (or hemi-) zygosity at specific genetic loci was recognised initially in retinoblastoma and subsequently in several other tumours (Knudson, 1971; 1985). The loci involved are believed to be sites of tumour suppressor genes or 'anti-oncogenes' which are relevant both to somatic events giving rise to sporadic tumours and to genetic predisposition to cancers (Lancet, 1988). In view of the potential importance of these issues for breast cancer screening programmes we have undertaken a survey of Ha-*ras* alleles in a cohort of 100 breast cancer patients.

### Patients and methods

Tumour and venous blood samples have been collected from 100 consecutive patients with histologically proven breast cancer, prior to any treatment (apart from the anti-oestrogen tamoxifen). All patients had presented with palpable breast lumps and were referred by their general practitioners to the breast clinic in the Royal Infirmary of Edinburgh. Patients with T4 tumours or with distant metastases at presentation, were excluded, as they are usually treated by chemotherapy in the first instance. The surgical procedures performed were either modified Patey mastectomy with axillary clearance, or wide local excision with axillary lymph node sampling. The resected specimen was immediately examined by the pathologist, tumour diameter measured in mm, and blocks taken for histological examination and for oestrogen receptor protein assay. The remainder was frozen on dry ice for later DNA extraction.

Lymph nodes were processed and examined for microscopic metastatic invasion. Tumours were classified into histological types as previously reported (Page & Anderson, 1988). Oestrogen receptor concentration was determined immediately by a saturation analytical method with separation of free and bound hormone using Dextran-coated charcoal adsorption as previously described (Hawkins *et al.*, 1981). Samples from patients who had received Tamoxifen were rechecked by enzyme immunoassay (Leclercq *et al.*, 1986). One hundred fresh placental samples have also been collected to act as a panel of normal controls representative of the local Edinburgh population. Permanent lymphoid cell lines were established from many of the blood samples by transformation *in vitro* with Epstein Barr virus. DNA was extracted from tumour and placental tissues and from blood and lymphoid cell lines (Steel, 1984). Ten $\mu g$ aliquots of genomic DNA were digested to completion with Bam Hl (Roberts *et al.*, 1977) (Boehringer, Mannheim GmbH), electrophoresed through 0.8% agarose, transferred to nylon membranes (Hybond, Amersham) and hybridised according to the manufacturer's instructions with the Harvey *ras* probe pEj (Shih & Weinberg, 1982), nick translated to a specific activity of $5 \times 10^7 - 1 \times 10^8$ cpm $\mu g^{-1}$ (Rigby *et al.*, 1977). After hybridisation, filters were washed at 65°C with 0.1XSSC (15mM Na Cl, 1.5mM $NA_3$ citrate, 0.1% Na PPi, 0.1% SDS) and exposed to Kodak X-Ar film at −70°C for 7–14 days, with intensifying screens. Similar procedures were followed with other probes and restriction enzymes as tabulated below.

### Results

### *Allelic frequency*

The four major c-Ha-*ras* Bam Hl alleles $A_1$–$A_4$, together with one rare variant $A'_1$ are shown in Figure 1.

Table I shows the relative frequencies of these alleles in blood and/or lymphoid cell line DNA from 100 breast cancer patients and in DNA from 100 placentae.

There was no significant difference between breast cancer

Correspondence: J. Mackay.
Received 11 May 1988; and in revised form, 11 July 1988.

**Figure 1** Alleles of c-Ha-*ras* (Bam HI digests) from four placental DNA samples. The left hand track of this Southern Blot contains a 'doublet' of allele $A_1$ and the rare variant $A'_1$.

**Table I** Bam HI alleles of Harvey *ras* locus

| | *100 Breast cancer patients* | | *100 Placentae* | |
|---|---|---|---|---|
| | *Number* | *%* | *Number* | *%* |
| $A_1$ | 126 | 63.0 | 135 | 67.5 |
| $A_2$ | 25 | 12.5 | 27 | 13.5 |
| $A_3$ | 23 | 11.5 | 19 | 9.5 |
| $A_4$ | 19 | 9.5 | 15 | 7.5 |
| Rare alleles[a] | 7 | 3.5 | 4 | 2.0 |

[a]$A'_1$ and $A''_1$... slightly smaller and larger respectively than $A_1$.

patients and controls in the frequencies of rare Harvey *ras* alleles; nor was there any shift in the distribution of common alleles between the two groups.

*Allele loss in tumours*

Complete or partial loss of a c-Ha-*ras* allele was established by comparing paired tumour DNA and white blood cell DNA samples from the same patients (Figure 2).

The one hundred tumours analysed fall into the following three categories. No allelic loss at the Ha-*ras* locus (51 tumours), loss of one allele (14 tumours) and uninformative, because the patient was constitutionally homozygous (35 tumours).

There was no preferential loss or retention of any of the four common alleles and our present analysis does not allow us to determine the maternal or paternal derivation of a deleted allele. We found no significant correlation between allelic loss and menopausal status, age, or history of an affected first degree relative. However, as shown in Table II, there was a significant correlation between loss of a Ha-*ras* allele and paucity of oestrogen receptor protein; absence of oestrogen receptor being a well-recognised index of poor prognosis (Croton *et al.*, 1981; Moore *et al.*, 1983; Williams *et al.*, 1987).

**Figure 2** Bam HI digests of tumour (T) and blood leukocyte (B) DNA from the same patient, probed with c-Ha-*ras* and compared with three placental controls. Note that alleles $A_1$ and $A_3$ are of equal intensity in B but allele $A_1$ is almost absent from the tumour sample.

There was also a significant correlation between tumour size and allelic loss as shown in Figure 3.

There was no significant correlation between allelic loss and pathological lymph node involvement, vascular invasion or histological type of tumour.

In order to assess the specificity of loss of the Harvey *ras* allele we have examined up to 5 other loci on the short arm of chromosome 11, comparing tumour DNA with lymphoblastoid cell line DNA from the same patient, as detailed in Table III.

Heterozygosity was found on a total of 49 occasions and the corresponding tumours had lost an allele in 19 cases (38.8%). Nineteen tumour/cell line pairs have been fully characterised for all 5 loci and allelic loss at one or more has been found in 10 (53%).

We have also studied one informative locus (pepsinogen) on the long arm of chromosome 11 and three at other chromosomal sites (5q, 6p and 17q). Of 67 instances where the patient was constitutionally heterozygous allele loss in the tumour was found outside of the 11p region on only one occasion (Table IV).

### Discussion

These results, in agreement with several published studies (Krontiris *et al.*, 1985; Heighway *et al.*, 1986; Thein *et al.*, 1986; Ceccherini-Nelli *et al.*, 1987; Gerhard *et al.*, 1987) demonstrate that rare Ha-*ras* alleles can be identified in the normal population. We have found no evidence for an increased frequency of rare alleles in breast cancer patients, contradicting both Krontiris' initial report (Krontiris *et al.*, 1985) and the findings of a subsequent larger series (Lidereau *et al.*, 1986) in which rare alleles were identified in 41% of breast cancer patients. Heighway *et al.* (1986) reported a preponderance of the A4 allele in patients with non-small-cell lung carcinoma, but several other studies have failed to find evidence of linkage in myelodysplasia (Thein *et al.*, 1986), colorectal adenocarcinoma (Hayward *et al.*, 1988) or familial melanoma (Gerhard, 1987). Lidereau's study on breast cancer patients was performed on breast tumour material which had been stored for up to 7 years, while the controls were fresh blood samples from unaffected individuals. Wyllie *et al.* (1988) has suggested that prolonged storage could lead to the identification of spurious 'rare' alleles, and we have therefore used DNA from white blood cells or lymphoblastoid cell lines, as well as tumour material.

In contrast to these negative findings, the observation that a substantial proportion of breast cancers have lost one c-Ha-*ras* allele confirms the recent report of Theillet *et al.* (1986) and lends some support to the hypothesis that the Ha-*ras* locus may be involved in breast cancer, albeit on a rather different theoretical basis.

Knudson's 'two hit' hypothesis (Knudson, 1971) provides a link between the molecular mechanisms underlying familial and sporadic forms of the same type of cancer. In sporadic cancer, a cell undergoes a somatic mutation, which must then be followed by a second event to express the malignant phenotype, either a second somatic mutation or loss of the unmutated allele by non-disjunction or deletion.

Following the localisation of the retinoblastoma gene to 13q14 (Cavenee *et al.*, 1983) comparable deletions at other sites have been reported in a variety of tumours, including Wilms' tumour (Koufos *et al.*, 1985), lung cancer (Kok *et al.*, 1987) and acoustic neuroma/meningioma (Seizinger *et al.*, 1986).

The present findings raise the question 'Is the reduction to homozygosity of the Harvey *ras* gene in breast cancer merely an indication that there has been a deletion somewhere on chromosome 11 and is there another gene in the region much more directly involved in the disease?' Ali and colleagues (1987) recently reported a total of 14 allele losses, distributed between five polymorphic loci on 11p in breast

**Table II** Relationship between loss of a c-Ha-*ras* allele and oestrogen receptor level in 61 breast tumours

| | *ER poor/-ve* $<20 fmol\,mg^{-1}$ *protein* | *ER moderate/rich* $\geqslant 20 fmol\,mg^{-1}$ *protein* (*20*) |
|---|---|---|
| Allelic loss | 8 | 6 |
| No allelic loss | 10 | 37 |

$P = <0.02$.

**Figure 3** Distribution of primary tumour size (greatest diameter mm) in relation to Ha-*ras* allele status.

**Table III** Details of allelic losses on 11p in breast tumour DNA compared with corresponding lymphoid cell line DNA

| *Tumour/ cell line pair* | *Ha*-ras **1* | *β-globin *2* | *PTH *3* | *Calcit *4* | *FSH-β *5,6* | *P11F9 *7,8* |
|---|---|---|---|---|---|---|
| 1 | a— | u | a— | a— | a— | a— |
| 2 | a— | a— | u | u | a— | u |
| 3 | a— | u | u | a— | u | u |
| 4 | a— | u | u | ab | ab | u |
| 5 | a— | u | u | u | u | ab |
| 6 | a— | u | u | u | u | ab |
| 7 | a— | ab | u | u | u | ab |
| 8 | a— | ab | a— | u | a— | ab |
| 9 | ab | u | u | u | u | a— |
| 10 | ab | u | u | u | u | ab |
| 11 | ab | ab | ab | u | u | ab |
| 12 | ab | ab | ab | u | u | ab |
| 13 | u | ab | u | u | ab | a— |
| 14 | u | ab | a— | u | u | a— |
| 15 | u | u | a— | u | u | a— |
| 16 | u | ab | u | u | u | ab |
| 17 | u | u | ab | u | ab | u |
| 18 | u | a— | u | u | u | ab |
| 19 | u | a— | u | u | u | ab |

ab = informative, not lost; u = uninformative; a — = allele loss.
'a' and 'b' should not be taken to refer to specific alleles.
*1 = Shih & Weinberg (1982); *2 = Deisseroth *et al.* (1978); *3 = Naylor *et al.* (1982); *4 = Höppener *et al.* (1984); *5,6 = Glaser *et al.* (1985), Watkins *et al.* (1985); *7,8 = Porteous *et al.* (1987), Boyd *et al.* (submitted).

cancers from 9 patients, not all of whom were analysed for every locus. We find reduction to hemizygosity of several sequences other than Harvey *ras* on the same chromosome arm, at least one locus being involved in 10 of 19 tumours (53%), a frequency even higher than the corresponding figure for Ha-*ras* (21.5%) and certainly much higher than for informative loci outside 11p (1 of 67 informative loci, in 27 breast tumours). It might be unwise to extrapolate from the present data, for example, to suggest that 11p deletions can be inferred in almost 50% of primary breast cancers since only 19 tumours have been analysed in detail so far and they include 8 already known to have lost a Ha-*ras* allele. Nevertheless there has clearly been a substantial frequency of DNA lesions within the short arm of chromosome 11 in our tumour material. The simplest interpretation is that a single mitotic recombination event has caused loss of all loci distal to the breakpoint which, in some instances, must have been on the centromeric side of the most proximal sequence examined, P11F9, at 11p13. At least three of the tumours studied, however, show patterns of allele loss incompatible with this simple mechanism since one or more loci on the telomeric side of the region of hemizygotisation remain heterozygous and in one case (No. 8, Table III) there were two regions of hemizygotisation separated by a locus that remains heterozygous. It is necessary, therefore, to invoke either multiple mitotic recombination events, localised chromosome deletions, partial inversions or even more complex rearrangements. More extensive mapping studies are required to resolve the issues raised by these observations and analysis is now being extended to cover all one hundred tumours in our series. One objective is to identify the smallest region of chromosome 11p that is consistently included in any deletion that can be mapped. Such a region might then be the site of a putative tumour suppressor gene (Friend *et al.*, 1988).

This of course does not preclude the specific involvement of other regions of the genome not examined in the present study and it is quite possible that two or more putative anti-oncogenes are involved in breast cancer (Lancet editorial, 1988). It is relevant to note that loss of heterozygosity has been reported on 13q in 6 out of 10 ductal breast cancers (Lundberg *et al.*, 1987).

We find that loss of a Ha-*ras* allele correlates with paucity of oestrogen receptor protein and with larger tumour size at presentation, but there is no correlation between pathological lymph node involvement and loss of a Ha-*ras* allele. Theillet *et al.* (1986) concluded that Ha-*ras* allelic loss was significantly linked to parameters of tumour aggressiveness since, in their material there were correlations between allelic loss and paucity of oestrogen receptor protein, histological grade and early occurrence of distant metastasis.

**Table IV**

| *Gene or probe* | *Designation* | *Localisation* | *Restriction enz* | *Ref*[a] | *No. examined* | *No. informative* | *No lost* |
|---|---|---|---|---|---|---|---|
| Pepsinogen | pH PEP | 11q12 | EcoR1 | 1,2 | 24 | 20 | 0 |
| Erb $A_2$ | pHeA2 | 17q21.3 | Bam Hl | 3 | 20 | 11 | 0 |
| MHC Class II | p11-B-4 | 6p21.3 | EcoR1 | 4 | 21 | 21 | 0 |
| λMS8 | D8S43 | 5q34qter | Hin Fl | 5,6 | 18 | 15 | 1 |
| | | | | | Total | 67 | 1 |

[a]1,2 = Taggart *et al.* (1985; 1987); 3 = Gosden *et al.* (1986); 4 = Gustafsson *et al.* (1984); 5,6 = Solomon *et al.* (1987), Wong *et al.* (1987).

Of the six breast tumours identified in this series as uninformative or heterozygous for c-Ha-*ras*, but showing loss of an allele at one or more loci elsewhere on 11p (cases 9, 13, 14, 15, 18 and 19, Table III), five had low or absent oestrogen receptor. The correlation between this prognostic feature and hemizygotisation of sequences somewhere on 11p therefore does not seem to be exclusive to the Harvey-*ras* locus. Although such a conclusion must be tentative until a larger number of tumours has been analysed in similar detail, the suggestion is that c-Ha-*ras* serves as a relatively inefficient index of hemizygotisation of a specific locus some distance away.

It will be of great importance to map that putative locus and thereafter to reassess the clinico-pathological correlations already established, to see if information of prognostic value can be obtained from DNA analysis of tumours at presentation. The clinical relevance of such findings will be established by follow up of our patient cohort to gather data on disease-free interval and long-term survival. Ultimately the objective is to define the gene itself and to establish the mechanism whereby it contributes to the evolution of breast cancer.

The authors are indebted to Prof Sir Patrick Forrest and Prof H.J. Evans for initiating this work and wish to thank them, and Dr T.J. Anderson and Dr W.R. Miller for their advice and encouragement, and Dr P.G. Middleton, Mr C de Angelis, Agnes Gallacher, Marie Robertson and the Obstetrics and Gynaecology Department of the Western General Hospital for their assistance. N. Davidson, A. Bruce and D. Stuart prepared the figures and Mrs Ann Kenmure typed the manuscript.

James Mackay holds a Margaret and Annie MacKenzie Scholarship from the Faculty of Medicine in the University of Edinburgh.

### References

ALI, I.U., LIDEREAU, R., THEILLET, C. & CALLAHAN, R. (1987). Reduction to homozygosity of genes on chromosome 11 in human breast neoplasia. *Science*, **238**, 185.

BOYD, P.A., CHRISTIE, S., HASTIE, N.D. & PORTEOUS, D.J. (0000). Rapid isolation of moderate and highly polymorphic DNA fragments mapping close to WT and AN2 on chromosome 11.

CAPON, D.J., CHEN, E.Y., LEVINSON, A.D., SEEBURG, P.H. & GOEDDEL, D.V. (1983). Complete nucleotide sequences of the T24 human bladder carcinoma oncogene and its normal homologue. *Nature*, **302**, 33.

CAVENEE, W.K., DRYJA, T.P., PHILLIPS, R.A. & 6 others (1983). Expression of recessive alleles by chromosomal mechanism in retinoblastoma. *Nature*, **305**, 779.

CECCHERINI-NELLI, L., DE RE, V., VIEL, A. & 4 others (1987). Ha-*ras*-1 restriction fragment length polymorphism and susceptibility to colon adenocarcinoma. *Br. J. Cancer*, **56**, 1.

CROTON, R., COOKE, T., HOLT, S., GEORGE, W.D. & 2 others (1981). Oestrogen receptors and survival in early breast cancer. *Br. Med. J.*, **283**, 1289.

DEISSEROTH, A., NIENHUIS, A., LAWRENCE, J., GILES, R., TURNER, P. & RUDDLE, F.H. (1978). Chromosomal localization of human $\beta$ globin gene on human chromosome 11 in somatic cell hybrids. *Proc. Natl Acad. Sci.*, **75**, 1456.

FRIEND, S.H., DRYJA, T.P. & WEINBERG, R.A. (1988). Oncogenes and tumor-suppressing genes. *N. Engl. J. Med.*, **318**, 618.

GERHARD, D., DRACOPOLI, N.C., BALE, S.J. & 5 others (1987). Evidence against Ha-*ras*-1 involvement in sporadic and familial melanoma. *Nature*, **325**, 73.

GLASER, T., GERHARD, D., JONES, C., ALBRITTAN, L., LALLEY, P. & HAUSMAN, D. (1985). A fine structure deletion map of chromosome 11p. *Cytogenet. Cell. Genet.*, **40**, 643.

GOSDEN, J.R., MIDDLETON, P.G., ROUT, D. & DE ANGELIS, C. (1986). Chromosomal localization of the human oncogene ERB A2. *Cytogenet. Cell. Genet.*, **43**, 150.

GUSTAFSSON, K., WIMAN, K., EMMOTH, E., LARHAMMAR, D., BOHME, J. & 4 others (1984). Mutations and selection in the generation of Class II histocompatibility antigen polymorphisms. *EMBO J.*, **3**, 1655.

HAWKINS, R.A., BLACK, R., STEELE, R.J.C., DIXON, J.M.J. & FORREST, A.P.M. (1981). Oestrogen receptor concentration in primary breast cancer and axillary node metastases. *Breast Cancer Res. Treat.*, **1**, 245.

HAYWARD, N.K., KEEGAN, R., NANCARROW, D.J., LITTLE, M.H. & 3 others (1988). c-Ha-*ras*-1 alleles in bladder cancer, Wilms' tumour and malignant melanoma. *Hum. Genet.*, **78**, 115.

HEIGHWAY, J., THATCHER, N., CERNY, T. & HASLETON, P.S. (1986). Genetic predisposition to human lung cancer. *Br. J. Cancer*, **53**, 453.

HÖPPENER, J.W.M., STEENBERGH, P.H., ZANDBERG, J. & 5 others (1984). Localisation of the polymorphic human calcitonin gene on chromosome 11. *Hum. Genet.*, **66**, 309.

KNUDSON, A.G. (1971). Mutation and cancer: Statistical study of retinoblastoma. *Proc. Natl Acad. Sci. USA.*, **68**, 820.

KNUDSON, A.G. (1985). Hereditary cancer: Oncogenes and anti-oncogenes. *Cancer Res.*, **45**, 1437.

KOK, K., OSINGA, J., CARRITT, B. & 9 others (1987). Deletion of a DNA sequence at the chromosomal region of 3p21 in all major types of lung cancer. *Nature*, **330**, 578.

KOUFOS, A., HANSEN, M.F., COPELAND, N.G. & 3 others (1985). Loss of heterozygosity in three embryonal tumours suggests a common pathogenetic mechanism. *Nature*, **316**, 330.

KRONTIRIS, T.G., DI MARTINA, N.A., COLB, M. & PARKINSON, D.R. (1985). Unique allelic restriction fragments of the human Ha-*ras* locus in leukocyte and tumour DNAs of cancer patients. *Nature*, **313**, 369.

LANCET editorial (1988). Molecular mechanisms in familial and sporadic cancers. *Lancet*, **i**, 92.

LECLERCQ, G., BOJAR, H., GOUSSARD, J. & 6 others (1986). Abbott monoclonal enzyme immunoassay measurement of estrogen receptors in human breast cancer: A European multicenter study. *Cancer Res.*, **46**, 4233s.

LIDEREAU, E., CHANTAL, E., THEILLET, C. & 8 others (1986). High frequence of rare alleles of the human c-Ha-*Ras*-1 proto-oncogene in breast cancer patients. *J. Natl Cancer Inst.*, **77**, 697.

LUNDBERG, C., SKOOG, L., CAVENEE, W.K. & NORDENSKJOLD, M. (1987). Loss of heterozygosity in human ductal breast tumours indicates a recessive mutation of chromosome 13. *Proc. Natl Acad. Sci. USA.*, **84**, 2372.

McBRIDE, O.W., SWAN, D.C., SANTOS, E., BARBACID, M., TRONICK, S.R. & AARONSON, S.A. (1982). Localization of the normal allele of T24 human bladder carcinoma oncogene to chromosome 11. *Nature*, **300**, 773.

MOORE, D.H., MOORE, D.U.T. & MOORE, C.T. (1983). Breast carcinoma etiological factors. *Adv. Cancer Res.*, **40**, 189.

NAYLOR, S.L., SAKAGUCHI, A.Y., KRONENBERG, H. & 4 others (1982). Human genomic organization of glycoprotein and polypeptide hormone genes. *Am. J. Human Genet. USA.*, **34**, 165A.

PAGE, D.L. & ANDERSON, T.J. (1988). *Diagnostic Histopathology of the Breast*. Churchill Livingstone.

PORTEOUS, D.J., BICKMORE, W., CHRISTIE, S., BOYD, P.A. & 8 others (1987). H-*Ras*-1 selected chromosome transfer generates markers that colocalise aniridia and genitourinary dysplasia-associated translocation breakpoints and the Wilms tumour gene within band 11p13. *Proc. Natl Acad. Sci. USA.*, **84**, 5355.

RIGBY, P.W.J., DIECKMANN, M., RHODES, C. & BERG, P. (1977). Labelling DNA to high specific activity *in vitro* by nick translation with DNA polymerase I. *J. Mol. Biol.*, **113**, 237.

ROBERTS, M.M., HAWKINS, R.A., ALEXANDER, F.E., ANDERSON, T.J. & STEELE, R.J.C. (1987). Oestrogen receptor activity in breast cancer detected at a prevalence screening examination. *Breast Cancer Res. Treat.*, **10**, 267.

ROBERTS, R.J., WILSON, G.A. & YOUNG, F.E. (1977). Recognition sequence of specific endonuclease Bam HI from Bacillus amyloliquefaciens H. *Nature*, **265**, 82.

SEIZINGER, B.R., MARTUZA, R.L. & GUSELLA, J.F. (1986). Loss of genes on chromosome 22 in tumorigenesis of human acoustic neuroma. *Nature*, **322**, 644.

SHIH, C. & WEINBERG, R.A. (1982). Isolation of a transforming sequence from a human bladder carcinoma cell line. *Cell*, **29**, 161.

SOLOMON, E., VOSS, R., HALL, V., BODMER, W.F., JASS, J.R. & 4 others (1987). Chromosome 5 allele loss in human colorectal carcinomas. *Nature*, **328**, 616.

STEEL, C.M. (1984). DNA in medicine: The tools. I and II. *Lancet*, **ii**, 908 and 966.

TAGGART, R.T., MOHANDAS, T.K., SHAWS, T.B. & BELL, G.I. (1985). Variable numbers of pepsinogen genes are located in the centromeric region of human chromosome 11 and determine the high frequency electrophoretic polymorphism. *Proc. Natl Acad. Sci. USA.*, **82**, 6240.

TAGGART, R.T., MOHANDAS, T.K. & BELL, G.I. (1987). Parasexual analysis of human pepsinogen molecular heterogeneity. *Somatic Cell and Molecular Genetics*, **13**, 167.

THEILLET, C., LIDEREAU, R. & ESCOT, C. (1986). Loss of a c-H-*ras*-1 allele and aggressive human primary breast carcinomas. *Cancer Res.*, **46**, 4776.

THEIN, S.L., OSCIER, D.G., FLINT, J. & WAINSCOAT, J.S. (1986). Ha-*ras* hypervariable alleles in myelodysplasia. *Nature*, **321**, 84.

WATKINS, P., EDDY, R., BECK, A., VELLUCCI, V., GUSELLA, J. & SHAWS, T. (1985). Assignment of the human gene for the beta subunit of follicle stimulating hormone (FSHB) to chromosome 11. *Cytog. and Cell Genet.*, **40**, 773.

WILLIAMS, M.R., TODD, J.H., ELLIS, I.O. *et al.* (1987). Osteogen receptors in primary and advanced breast cancer. An eight year review of 704 cases. *Br. J. Cancer*, **55**, 67.

WONG, Z., WILSON, V., PATEL, I., POVEY, S. & JEFFREYS, A.J. (1987). Characterization of a panel of highly variable minisatellites cloned from human DNA. *Ann. Hum. Genet.*, **51**, 269.

WYLLIE, F.S., WYNFORD-THOMAS, U., LEMOINE, N.R., WILLIAMS, G.T. *et al.* (1988). Ha-*ras* restriction fragment length polymorphisms in colorectal cancer. *Br. J. Cancer*, **57**, 135.